# Retraction: Magnetic Properties of Polyvinyl Alcohol and Doxorubicine Loaded Iron Oxide Nanoparticles for Anticancer Drug Delivery Applications

**DOI:** 10.1371/journal.pone.0337985

**Published:** 2025-12-02

**Authors:** The PLOS One Editors

After this article [1] was published, concerns were raised about Figs 3, 5 and 7.

Specifically:

- In Fig 3, there appear to be repetitive elements within and between the XRD patterns for each sample.
- In Figs 5b and 5c, there appear to be similarities in the EDS spectra between 1-6 keV.
- In the Fig 7c TEM panel, similarities were noted between multiple regions in the background and within each of the IONPs complexes.

The first and corresponding author MN stated that the apparent similarities within the XRD patterns of Fig 3 are because the samples share the same crystalline phase. Regarding Fig 5, they stated that both EDS spectra correspond to samples with a common iron-oxide core, and the spectral resemblance is an expected physical outcome. They stated that the similarities observed within Fig 7C were due to the uniformity of the synthesized nanoparticles and imaging contrast settings. The first author stated that all data underlying these figures are no longer available. As such, PLOS does not consider these concerns to be resolved.

In light of the above unresolved concerns that question the reliability and integrity of the reported results, the *PLOS One* Editors retract this article.

MN, MSA, SR, and MAS did not agree with the retraction. MA, AS, SN, and MM either did not respond directly or could not be reached.
